# The Classical–Basal Spectrum in Pancreatic Ductal Adenocarcinoma: A Developmental Framework for Tumour Cell Plasticity and Clinical Translation

**DOI:** 10.3390/cancers18172733

**Published:** 2026-08-23

**Authors:** Ikra Khan, Azzadinne Belhaj, Alessandro Gemini, Kenza Azra Ibis, Nouman Darsif, Najoua Rouani, Aude Vanlander, Nouredin Messaoudi

**Affiliations:** Department of Hepatopancreatobiliary Surgery, Vrije Universiteit Brussel, Universitair Ziekenhuis Brussel and Europe Hospitals, 1090 Brussels, Belgium; ikrakhan@vub.be (I.K.); azzadinne.belhaj@vub.be (A.B.); alessandro.gemini@hotmail.it (A.G.); kenza.azra.ibis@vub.be (K.A.I.); nouman.darsif@vub.be (N.D.); n.rouani@cdle.be (N.R.); aude.vanlander@uzbrussel.be (A.V.)

**Keywords:** pancreatic ductal adenocarcinoma, molecular subtypes, basal-like, classical, GATA6, tumour plasticity, cancer-associated fibroblasts, spatial transcriptomics, digital pathology, precision oncology

## Abstract

Transcriptomic profiling reproducibly resolves pancreatic ductal adenocarcinoma (PDAC) into two prognostically divergent tumour-intrinsic programmes—classical/progenitor and basal-like/squamous—accompanied by a separable stromal axis; additional proposed classes are strongly influenced by low tumour cellularity and generalise poorly across datasets. Rather than cataloguing these programmes, this review synthesises evidence that they behave as plastic cell states and proposes an integrative developmental framework for interpreting that plasticity, rather than claiming its discovery. The framework combines a normal-pancreas developmental reference frame, the regulation of state by lineage transcription factors, epigenetic remodelling, oncogenic (KRAS) dosage, stromal instruction, and treatment pressure, and a testable notion of spatial disorganisation. Throughout, we separate robust prognostic evidence—basal-like assignment and low GATA6 expression are consistently associated with worse outcome—from predictive claims: predictive utility remains unproven because no completed prospective trial has assigned treatment according to classical–basal subtype and demonstrated improved clinical outcome from that strategy, and existing signals derive from retrospective or observational analyses. The proposition that the classical–basal spectrum reflects a spatially disordered redeployment of the normal duct’s luminal–basal hierarchy is presented as a hypothesis requiring validation in independent atlases rather than as established biology. We define the falsifiable experiments needed to test the framework and its clinical implications.

## Simple Summary

Pancreatic ductal adenocarcinoma is among the most lethal cancers, and most patients receive similar chemotherapy regardless of tumour biology. Molecular profiling reproducibly separates these tumours into two transcriptional groups: a better-prognosis “classical” group and an aggressive “basal-like” group. This distinction is well established and prognostic, but it does not yet reliably indicate which drug an individual patient should receive. In this review we summarise the biology of these two groups and argue that they are better viewed as two ends of a changeable spectrum than as fixed categories. We then set out an integrative framework linking this spectrum to normal pancreatic-duct biology, oncogene signalling, the surrounding tissue, and prior treatment, and we propose spatial disorganisation as a measurable feature worth testing. We separate what is established from what is proposed: the developmental interpretation and the spatial-disorder concept are hypotheses requiring independent validation, and no molecular subtype currently justifies changing treatment or surgical decisions outside a clinical trial.

## 1. Introduction

Pancreatic ductal adenocarcinoma (PDAC) is projected, on incidence and mortality modelling, to become the second leading cause of cancer-related death in the United States within the decade [1]; relative five-year survival remains approximately 13% for pancreatic cancer overall, with the adenocarcinoma subset faring worse [2]. For only 15–20% of patients present with resectable disease, surgery is the only potentially curative modality, and systemic therapy is still selected largely by performance status. Modified FOLFIRINOX is the adjuvant standard after resection [3]; FOLFIRINOX-based therapy and gemcitabine/nab-paclitaxel are used in advanced disease and selected neoadjuvant settings; and the liposomal-irinotecan regimen NALIRIFOX is a first-line metastatic option, having improved median overall survival from 9.2 to 11.1 months over gemcitabine/nab-paclitaxel in NAPOLI-3 [4]. None of these choices is currently guided by tumour biology—a gap that molecular subtyping was expected to close.

This review concerns the molecular (transcriptional) subtypes of conventional PDAC—principally the classical and basal-like programmes—rather than the recognised histological variants of pancreatic carcinoma. We use “subtype” for tumour-level classification, “cell state” for the plastic entity that varies within and between tumours, and “programme” for the transcriptional circuit that specifies a given state. Existing reviews largely describe these subtypes and catalogue their molecular features; this review asks a different question—what biological framework best explains why the classification behaves as a spectrum rather than a clean dichotomy? The classical–basal distinction is robust and prognostic, but discrete labels have proven difficult to translate into treatment selection, and we argue that this difficulty follows from the underlying biology: the two programmes behave as plastic cell states rather than fixed categories. Because plasticity and the classical–basal continuum are by now widely recognised, our aim is not to claim their discovery but to integrate them into a single developmental framework—one that links the spectrum to a normal-tissue reference frame, regulation by oncogenic dosage, epigenetic remodelling, stromal signals, and treatment pressure, and that introduces spatial disorganisation as a testable variable. A more specific proposition within this framework—that the spectrum reflects a spatially disordered redeployment of the normal duct’s luminal–basal hierarchy—is advanced as a hypothesis requiring independent validation; this point is stated here so it need not be repeated throughout.

## 2. Literature Search Strategy

For this review, we searched PubMed/MEDLINE from database inception, with the search last updated on 18 July 2026, combining terms for pancreatic cancer with concepts including molecular subtype, classical, basal-like, squamous, GATA6, single-cell, spatial transcriptomics, cancer-associated fibroblast, plasticity, cell of origin, digital pathology, and radiomics. Additionally, we hand-searched the reference lists of major primary studies and reviews. We prioritised primary landmark studies, prospective trials with translational analyses, and single-cell and spatial datasets, with emphasis on the past five years alongside seminal earlier work; selection was based on relevance and study quality by author consensus. No formal screening flow, risk-of-bias assessment, or quantitative synthesis was performed. Studies were included if they reported primary data on PDAC transcriptional subtypes, their regulation, spatial organisation, or clinical association, or if they were trials with subtype-relevant translational analyses; single-case reports, non-PDAC studies, and conference abstracts without peer-reviewed publication were not used to support substantive claims. Where several studies addressed the same point, priority was given to primary over secondary sources, to larger and prospectively collected cohorts, and to studies reporting purity-aware or spatially resolved data. Because study selection was purposive rather than exhaustive, the possibility of selection bias cannot be excluded, and this review should not be read as a systematic assessment of the totality of the evidence.

## 3. Convergence and Limitations of Existing Classification Systems

Independent transcriptomic studies converge on a two-pole tumour-intrinsic axis. Collisson and colleagues first proposed classical, quasi-mesenchymal, and exocrine-like subtypes from cell lines and microdissected tissue [5]; Moffitt and colleagues used computational microdissection to separate tumour-intrinsic classes (classical, basal-like) from stromal classes [6]; Bailey and colleagues described four genomic subtypes, adding immunogenic and aberrantly differentiated endocrine–exocrine (ADEX) categories [7]. The integrated TCGA analysis, which included low-cellularity specimens, showed that the ADEX, exocrine-like, and immunogenic signatures arise predominantly in samples with few neoplastic cells and are strongly influenced by non-neoplastic admixture rather than representing tumour-cell-autonomous programmes [8]. Whole-tissue and single-nucleus schemes subsequently recovered groups that are best understood as combinations of the tumour and stromal axes, including tumours co-expressing both programmes [9,10]. Across schemes, the squamous, quasi-mesenchymal, and basal-like designations correspond approximately, as do progenitor and classical (Table 1); these mappings are approximate rather than exact equivalences. The principal molecular, morphological, and clinical features distinguishing the two poles are summarised in Table 2.

Two observations indicate that much of the residual disagreement is technical. A purity-independent single-sample classifier based on within-sample gene-pair rankings (PurIST) provides a reproducible two-class representation of the classical–basal spectrum across platforms, whereas finer schemes do not [11]; this two-class output is a practical simplification, not evidence that the underlying biology is strictly binary. Independently, a systematic re-evaluation found that widely used multi-gene signatures lack cross-dataset reproducibility, with permuted subtypes and random gene sets achieving comparable performance and with sample-preparation and purity effects dominating [12]. The reasonable interpretation is that the two-pole classical–basal spectrum is a robust biological observation, while forced binary assignment loses intermediate and intratumoural information; the additional classes largely reflect admixture and analytic choices. This has a practical corollary: any deployable assay must be purity-aware.

## 4. The Classical–Basal Axis: Discrete Subtypes, Continuous States, or Attractors?

At bulk resolution the axis appears bimodal, but higher-resolution data are compatible with several models. In patient-derived organoids and primary tumours, classical and basal-like cells coexist and are connected by a differentiation hierarchy, with classical cells enriched at a differentiated endpoint [13]. Multiplex imaging shows basal–classical co-expressor cells in most tumours, occupying an intermediate position, and a continuous polarisation score associated with survival [14]. Three interpretations remain consistent with these observations: a predominantly continuous spectrum of states; two relatively stable poles separated by a populated intermediate region; and context-dependent, attractor-like behaviour in which cells settle into recurrent configurations. Cross-sectional human datasets cannot distinguish definitively among these, because they capture single time points rather than trajectories. “Attractor” is therefore used here only as a conceptual interpretation; formal dynamical or perturbation studies would be required to establish it. What the data do support is that intermediate cells are biologically real rather than measurement noise, which reframes the “hybrid” tumours of single-nucleus studies and the co-expressor cells of imaging studies as occupied middle ground [10,14].

## 5. Molecular and Epigenetic Regulators of Cell State

The position along the axis is governed by competing lineage transcription factors and their enhancer landscapes (Table 2). The classical programme is specified by GATA6, HNF1A, HNF4A, and PDX1, sustaining epithelial differentiation and a lipogenic metabolic bias [7,8]. The basal programme is driven by ΔNp63 (TP63), which is sufficient to install and maintain the squamous enhancer landscape [15], integrated with squamous features into a unifying transcriptional account [16]. These programmes are organised into a mutually repressive network in which GATA6, the HNF factors, and ΔNp63 antagonise one another, providing a plausible basis for a tunable state and for the plasticity observed experimentally [17]. Epigenomic reprogramming links this identity to metabolic rewiring and metastatic competence, indicating that the state is chromatin-encoded rather than a passive transcriptional readout [18].

The role of KRAS is genuinely unsettled. In genetically engineered models, increased mutant-KRAS gene dosage drives dedifferentiation toward aggressive phenotypes [19], and in a clinicogenomic cohort of 2336 patients, KRAS mutant-allele dosage gains—reported in approximately one-fifth of KRAS-mutated, copy-number-diploid tumours—were prognostic across stages [20]; the COMPASS programme reported enrichment of major mutant-KRAS imbalances among basal-like tumours [21,22]. Other functional data, however, suggest that differentiated classical cells retain strong dependence on KRAS. Whether KRAS dosage principally drives cells toward the basal pole, or whether classical cells remain more KRAS-dependent, is not resolved, and the two possibilities carry opposite therapeutic implications. In situ analysis of resected tumours reported that GATA6 and mutant-KRAS^G12D protein are anti-correlated within individual ducts in a manner not explained by copy number [23], consistent with, but not establishing, a regulatory rather than purely genomic relationship.

## 6. Developmental and Normal-Tissue Reference Frames

The classifications discussed above are derived entirely from tumour data. Their poor cross-dataset generalisability has several technical causes—platform, sample preparation, tumour purity, cohort composition, and classifier construction [12]—which an external biological reference frame may help interpret but cannot by itself resolve. Such a reference also requires conceptual care: three notions must be kept distinct—the cell of origin (the initiating cell), resemblance to a normal cell type (transcriptional similarity), and a co-opted differentiation programme (a normal regulatory circuit reused by cancer)—since transcriptional similarity does not by itself establish lineage derivation. Both acinar and ductal cells can undergo malignant transformation experimentally, and the cell of origin can influence the resulting subtype [24], although the acinar origin, via acinar-to-ductal metaplasia, is more strongly supported in commonly used mouse models.

The evidence bearing on origin is mixed. Epigenomic data in human tumours associate an aggressive, interferon-active phenotype with a ductal cell-of-origin signature and an indolent phenotype with acinar-derived programmes [25], while ductal origin remains less firmly established than acinar origin in mouse models [25,26]. Against this background, recent work mapped a luminal–basal organisation in the normal human duct—including ΔNp63-positive basal cells and a distinct supra-basal luminal population—and reported that these native signatures resemble basal-like PDAC and associate with shorter survival, with ΔNp63 promoting a basal state [26,27,28]. That primary work frames these populations as potential transcriptional templates that cancer may co-opt rather than as demonstrated cells of origin, and explicitly notes that transcriptional similarity does not establish a lineage relationship [27]—a position we adopt here. The normal duct therefore serves as a developmental reference frame and a source of normal-lineage resemblance, not as evidence that the tumour spectrum directly reproduces the normal hierarchy or that these cells initiate PDAC. The framework’s central, testable prediction is that markers derived from normal lineage should classify tumours more robustly than tumour-derived signatures; confirming this requires replication of the normal-duct map in independent single-cell and spatial atlases, an independent validation these observations still await.

## 7. Spatial Heterogeneity and the Proposed Spatial-Disorder Model

Heterogeneity in PDAC is spatially structured. Within single ducts, classical and basal programmes are intermixed at single-cell resolution, with abrupt local transitions [23]; in metastatic disease, spatial mapping localises TGFB1-expressing myofibroblastic fibroblasts adjacent to basal-like tumour cells [29]; and single-cell and spatial technologies have refined the molecular and microenvironmental framework of the disease [30]. Notably, adenosquamous carcinoma segregates its glandular and squamous components into ordered domains, whereas conventional PDAC re-expresses comparable programmes in a fragmented spatial pattern [27], a contrast that motivates the spatial-disorder hypothesis developed below.

These observations motivate a hypothesis: that the degree of spatial disorder—how far a tumour’s lineage programme organisation departs from ordered tissue architecture—may itself be biologically informative. A spatial-disorder metric might quantify spatial entropy, fragmentation of lineage domains, neighbourhood mixing of classical and basal cells, loss of normal radial or basal–luminal organisation, spatial autocorrelation of program markers, or the distance between basal-like cells and specific fibroblast populations. Any such metric would be confounded by tumour grade, gland formation, necrosis, tumour cellularity, sampling area, segmentation accuracy, platform resolution, and treatment status, all of which would need to be controlled.

A framework for evaluating such a metric can nevertheless be pre-specified, and we set one out here so that the proposal is falsifiable rather than merely suggestive. First, a single primary metric should be nominated in advance—neighbourhood mixing of classical and basal-like cells within a defined radius is the most tractable candidate—with the remaining measures treated as secondary. Second, the platform and tissue requirements should be fixed: multiplex immunofluorescence or imaging-based spatial transcriptomics on whole sections rather than tissue microarrays, with sufficient field of view to capture both tumour centre and invasive front. Third, cell segmentation and phenotype calling should follow a locked, published pipeline, since segmentation error propagates directly into any neighbourhood statistic. Fourth, the metric should be analysed continuously, with any threshold derived in a training cohort and then fixed before testing. Fifth, technical reproducibility should be established on replicate sections and, separately, across at least two platforms, because a measure that is not stable between assays cannot become a biomarker. Sixth, the metric should then be tested in an independent external cohort against disease-free and overall survival, with adjustment for stage, grade, resection margin and treatment. Finally, and most importantly, it must be compared directly with conventional classical–basal classification: unless spatial disorder adds prognostic information beyond subtype fraction alone, it does not justify the additional assay burden. Until these steps are completed, spatial disorder remains a hypothesis about tumour organisation, not a biomarker.

## 8. Microenvironmental Regulation of Tumour State

Cancer-associated fibroblasts (CAFs) are heterogeneous and interconvertible, comprising myofibroblastic, inflammatory, and antigen-presenting populations [31,32]. The relationship between CAF composition and tumour-cell state is increasingly viewed as instructive rather than incidental: microenvironmental context alters malignant cell state and drug response in organoid systems [33], and spatial data place specific fibroblast populations near basal-like cells [29]. These associations should not be overgeneralised -much of the spatial evidence derives from particular cohorts and disease sites—but they indicate that the niche can modulate state. The desmoplasia paradox reinforces that stromal effects are context-dependent: genetic depletion of α-smooth-muscle-actin-positive myofibroblasts worsened outcomes in mouse PDAC, contradicting the assumption that fibrosis is uniformly pro-tumoural [34]. The evidence supports microenvironmental modulation of state; it does not establish that the niche determines state, since no study has shown that withdrawing a niche signal reverts an established basal tumour cell to a stable classical identity in vivo. The immune compartment is part of the same instructive niche and carries independent prognostic weight. Classical tumours with high GATA6 expression have been shown to be immune-enriched and to fare better after upfront surgery [35], and enhanced antitumour immunity following neoadjuvant chemoradiotherapy has been associated with more favourable prognosis in resected disease, particularly in women [36]. Read alongside the treatment-associated remodelling discussed in Section 9, these findings indicate that the immune context both covaries with tumour cell state and is itself modified by therapy, which is a further reason why a single pre-treatment subtype call is an incomplete description of a tumour.

## 9. Treatment-Induced Selection and Reprogramming

Subtype composition differs between treated and untreated tumours, but because human tissue is sampled cross-sectionally these comparisons cannot distinguish selection of pre-existing populations from genuine reprogramming. Single-nucleus and spatial profiling of treated tumours identified additional malignant programmes, including a neoadjuvant-associated state that the authors of that study termed neural-like progenitor [37]; GATA6’s prognostic discrimination is attenuated after gemcitabine-based chemoradiotherapy [35]; and intensified neoadjuvant therapy has been associated with histo-molecular remodelling, including a shift in tumour–stroma balance [38]. The distinction matters therapeutically: if selection dominates, subtype-directed therapy should be given up front; if reprogramming dominates, blocking the responsible signal becomes rational. Resolving it will require paired longitudinal sampling, lineage barcoding in patient-derived models, and analysis of residual disease—settings in which surgical cohorts can contribute.

## 10. Prognostic Evidence

Basal-like assignment and low GATA6 expression are consistently associated with a worse outcome across cohorts, platforms, and disease stages [8,10,21,39]. This prognostic signal is among the most reproducible findings in the field, but it is partly confounded: basal-like tumours present at a more advanced stage and with lower resectability, so some of the association reflects stage. The magnitude of the association also varies with the assay and the treatment context. These caveats do not undermine the prognostic validity of the axis; they bound its interpretation (Table 3).

## 11. Predictive Evidence and Current Clinical Limitations

A biomarker is predictive only if it identifies patients who benefit differentially from a specific therapy, requiring a statistically credible treatment-by-biomarker interaction or prospective biomarker-directed allocation; differential outcomes between molecular groups receiving different therapies do not, by themselves, establish this. By this standard, current evidence remains limited and hypothesis-generating. In the prospective but non-randomised COMPASS programme, basal-like tumours showed lower response rates and shorter survival on first-line chemotherapy—an association, not a demonstrated interaction [21,22]. The GemPred signature, developed to predict adjuvant gemcitabine sensitivity and not identical to the classical–basal spectrum, was evaluated in a retrospective ancillary analysis of PRODIGE-24/CCTG-PA6: in the GemPred-positive subgroup (approximately one-quarter of patients), observed survival estimates on gemcitabine were similar to those on modified FOLFIRINOX, with lower toxicity. This did not establish equivalence or non-inferiority, no formal treatment-by-biomarker interaction was tested, and the finding remains retrospective and hypothesis-generating pending prospective validation [40]. GATA6 immunohistochemistry is reproducibly prognostic in treatment-naïve resected disease, though its discrimination is attenuated after neoadjuvant chemoradiotherapy [35]; earlier work reported more rapid progression in patients with GATA6-low tumours treated with modified FOLFIRINOX but not gemcitabine—again an association, not a validated predictive effect [43]. A dedicated systematic review concluded that current evidence is insufficient to guide treatment or surgical decisions by subtype [47]. PASS-01 prospectively integrated whole-genome/transcriptome sequencing and organoid profiling within a randomised comparison of modified FOLFIRINOX and gemcitabine/nab-paclitaxel; it demonstrated the feasibility of intensive molecular profiling, but progression-free survival was similar between arms, treatment was not assigned by subtype, and no validated classical–basal treatment interaction emerged [41]. In short, no completed prospective trial has assigned treatment by classical–basal subtype and shown improved outcome from doing so, so te predictive utility remains unproven even though the spectrum is reliably prognostic. It is instructive to contrast this with candidate predictive biomarkers evaluated outside the classical–basal framework. Within COMPASS, hENT1 expression was associated with response and survival specifically among patients receiving gemcitabine/nab-paclitaxel and not among those receiving modified FOLFIRINOX, with a formal interaction test supporting a predictive rather than purely prognostic effect [48]; notably, hENT1 expression was itself higher in classical tumours, so this marker is correlated with, but not equivalent to, transcriptional subtype. Class III β-tubulin (TUBB3) has similarly been examined as a determinant of taxane sensitivity, and a combined analysis of TUBB3 and hENT1 expression in advanced PDAC reported both prognostic and treatment-related associations for these markers [49]. These single-gene markers are mechanistically anchored to a specific drug, which is precisely why an interaction can be demonstrated for them, whereas the classical–basal spectrum is a global differentiation state that plausibly influences sensitivity to several agents at once and is correspondingly harder to convert into a drug-specific predictive claim. Their existence does not diminish the subtype framework, but it does clarify what the framework must still achieve.

## 12. Assays and Sampling Considerations

Assays should be judged on three distinct criteria: analytical validity (technical reproducibility), clinical validity (association with outcome), and clinical utility (evidence that using the assay improves outcomes). No PDAC subtyping assay has demonstrated clinical utility, and none is an established clinical tool; all are research-grade or, at most, prognostic aids under evaluation (Table 3). Bulk RNA-sequencing supports discovery but is purity-sensitive [12]; PurIST gives a purity-independent two-class call [11]; PAMG gives a continuous score [42]; GATA6 immunohistochemistry is a pragmatic surrogate with demonstrated clinical validity but unproven utility [21,35].

Computer-assisted GATA6 scoring improved inter-reader concordance in one advanced-disease cohort while preserving the outcome association [43], and combination immunohistochemistry (e.g., CK5/6, p63, GATA6, HNF4A) recovers the two-pole distinction in treatment-naïve tumours [46]. Single-cell and spatial methods, liquid biopsy, and deep-learning inference from histology or imaging (pathomics and radiomics) remain research tools requiring external validation. Three sampling problems follow directly from this biology: purity, since stroma-rich fine-needle biopsies bias bulk classifiers toward admixture-driven calls [8,12]; representativeness, since a single core reports only a local fraction, though biopsy-scale tissue can recover representative fractions in some series [14]; and dynamism, since a baseline call may not survive treatment (Section 9). Turnaround times depend on platform and laboratory.

## 13. Implications for Surgery and Multimodal Treatment

Subtype should not currently influence resectability assessment, exploration thresholds, or surgical candidacy, and it should not alter surgical selection outside a clinical trial. The legitimate surgical contributions are to the evidence base rather than to intraoperative decisions. Surgeons and interventional endoscopists determine the quality and cellularity of the diagnostic material on which every assay depends, which matters increasingly as neoadjuvant treatment shifts diagnosis onto small, stroma-rich biopsies. Surgical and neoadjuvant pathways also generate paired pre- and post-treatment tissue, multi-region sampling, and standardised handling, enabling study of treatment-induced change and correlation of subtype with pathological response and residual disease. Embedding molecular and computational subtyping into neoadjuvant and surgical trials, with longitudinal tissue banking, is the principal way surgery can advance this field.

### Current Treatment of PDAC and Why Subtype Does Not Presently Alter It

Because this review argues for a dynamic reading of tumour state, it is worth stating plainly what current treatment consists of, and why the classical–basal spectrum does not yet modify any of it. For anatomically resectable disease, surgery remains the only potentially curative modality, and adjuvant modified FOLFIRINOX is the standard of care after resection in fit patients [3]. Neoadjuvant strategies are increasingly used in borderline resectable disease. In advanced disease, FOLFIRINOX-based regimens and gemcitabine/nab-paclitaxel remain the principal first-line options, with NALIRIFOX an additional first-line choice in the metastatic setting [4]; regimen selection is currently governed by performance status, comorbidity, and toxicity profile rather than tumour biology [50]. Molecularly targeted options exist but are defined by genomic rather than transcriptional features: they apply to small biomarker-defined subgroups such as BRCA-associated disease, mismatch-repair-deficient tumours, and rare fusion-positive cancers, and, prospectively, to KRAS-directed agents (Section 14) [50].

Against this background, the reason subtype does not currently alter the choice between FOLFIRINOX, gemcitabine/nab-paclitaxel, NALIRIFOX, neoadjuvant therapy or surgery is a matter of evidence level rather than of biological plausibility. Six distinct standards must be separated. A prognostic association means outcome differs by marker status irrespective of treatment; this is established for the classical–basal spectrum. A treatment association means outcome differs by marker status among patients who happened to receive a given therapy; this is what COMPASS and the GATA6 series report, and it is confounded by the reasons patients received that therapy. A predictive biomarker requires that the marker identify differential benefit from one therapy over another. Demonstrating this requires a treatment-by-biomarker interaction that is statistically credible and pre-specified, ideally within a randomised comparison. Beyond that, prospective biomarker-directed allocation requires that treatment actually be assigned by the marker. Clinical utility, finally, requires evidence that allocating treatment by the marker improves patient outcomes relative to not doing so. The classical–basal spectrum currently satisfies the first standard, partially satisfies the second, and has not been shown to satisfy the third, fourth, fifth or sixth.

What would change practice is therefore specifiable. The minimum requirement is a prospective randomised trial in which subtype is measured before treatment using a locked, purity-independent assay, patients are allocated to competing regimens either by subtype or with subtype as a pre-specified stratification factor, and the primary analysis tests the subtype-by-treatment interaction rather than outcome within subtype groups. Such a trial would need to be powered for the interaction, not merely for the main effect, which is a substantially larger requirement. PASS-01 embedded prospective molecular and organoid profiling within a randomised comparison of modified FOLFIRINOX and gemcitabine/nab-paclitaxel and demonstrated that such profiling is feasible in a multicentre setting, but treatment was not allocated by subtype and no validated classical–basal interaction emerged [41]. Until a trial of this design reports, subtype-directed treatment remains a research strategy, and neither systemic-therapy selection nor surgical decision-making should be altered by subtype outside a clinical trial.

## 14. KRAS-Directed Therapy: Evidence and Testable Hypotheses

The clinical arrival of KRAS-directed agents makes the relationship between subtype and KRAS dependence therapeutically relevant. Selective non-covalent KRAS-G12D inhibitors, exemplified by MRTX1133, show anti-tumour activity in preclinical PDAC models, including immunocompetent systems with evidence of microenvironmental remodelling [44,45,51]; pan-RAS and additional G12D-selective agents are in clinical development. Clinical data in PDAC remain in its early stages.

Whether subtype predicts response is an open question with competing, testable hypotheses rather than a settled expectation. At least four possibilities merit consideration: that higher mutant-KRAS dosage in basal-like tumours increases their vulnerability to KRAS inhibition [19,20]; that basal-like tumours instead depend more on bypass signalling, epigenetic plasticity, or alternative pathways and are therefore more resistant; that classical, epithelial tumours retain greater lineage dependence on KRAS and respond better; and that microenvironmental and immune effects influence response independently of tumour-cell subtype. These are not resolved by existing data, and there is no direct evidence that KRAS inhibition reprograms basal-like cells toward a classical state; any such lineage shift should be treated as an exploratory, measurable endpoint in subtype-annotated trials rather than an expected outcome.

## 15. Proposed Framework for Clinical Translation

**Figure 1:**
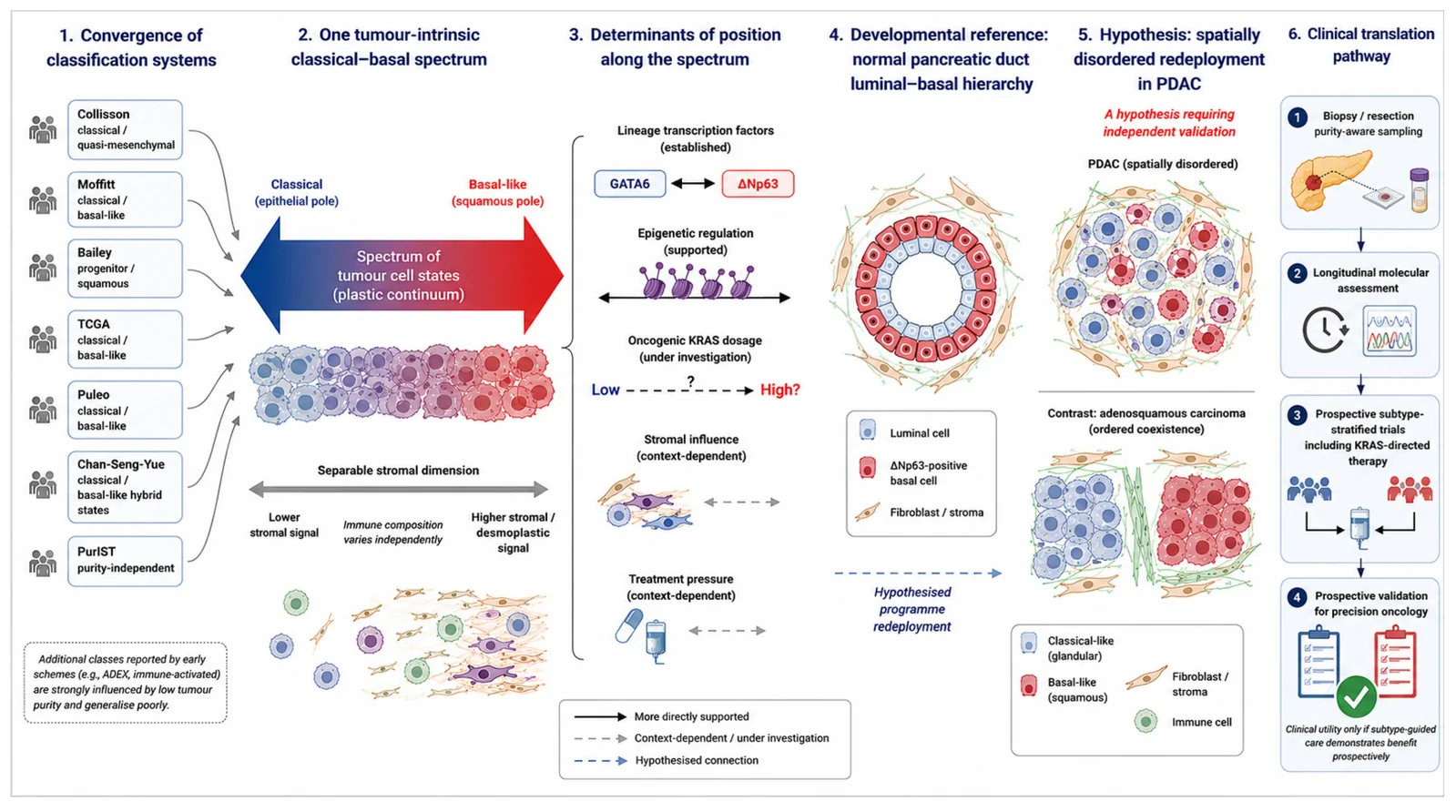
Integrated developmental and translational framework for the classical–basal spectrum in pancreatic ductal adenocarcinoma. Independent transcriptomic classification systems (1) converge on a tumour-intrinsic continuum extending from a classical epithelial state to a basal-like squamous state, with intermediate states populated and cell-state position best regarded as plastic rather than fixed; PurIST provides a purity-independent classification, and additional classes described in earlier schemes are strongly influenced by low tumour purity and non-neoplastic admixture. A separable stromal dimension (2) accompanies this tumour-intrinsic spectrum, together with an immune composition that is not fully explained by tumour-intrinsic state. Position along the classical–basal spectrum (3) is governed by opposing lineage transcriptional programmes centred on GATA6 and ΔNp63, supported by epigenetic regulation, whereas the contributions of oncogenic KRAS dosage, stromal influence and treatment pressure remain context-dependent or incompletely resolved. The normal pancreatic duct (4), in which luminal cells are surrounded by ΔNp63-positive basal cells, is presented as a developmental reference frame rather than a demonstrated cell of origin. We hypothesise (5) that PDAC may represent a spatially disordered redeployment of these lineage programmes, in contrast to the more ordered coexistence of glandular/classical-like and squamous/basal-like domains in adenosquamous carcinoma; this model requires independent validation. Clinical translation (6) would require purity-aware sampling, longitudinal molecular assessment and prospective subtype-stratified trials, including evaluation in the context of KRAS-directed therapy; implementation in precision oncology should follow only if prospective studies demonstrate clinical utility of subtype-guided care. Solid, grey dashed and blue dashed arrows denote more directly supported relationships, context-dependent or unresolved relationships, and hypothesised connections, respectively.

This is a research framework, not a clinical guideline: no element should alter standard care outside a trial (Figure 1). Four principles distinguish it from current practice: reporting position along the classical–basal spectrum, and where feasible an exploratory spatial-disorder metric, rather than a binary label [14,42]; using purity-independent, computational assays for small biopsies, validated against a normal-lineage reference where possible [11,43,46]; reassessing subtype longitudinally through therapy rather than treating it as fixed (Section 9); and designing subtype-adaptive trials, including correlative testing of whether KRAS-directed or chromatin-directed therapy shifts cell state (Section 11 and Section 14). The framework is falsifiable: if lineage-grounded, spatially aware, longitudinal classification fails to outperform binary baseline subtyping in a prospective predictive setting, it should be discarded.

## 16. Research Priorities

Priorities follow from the preceding sections (Table 3): replication of the normal-duct luminal–basal map in independent atlases; experimental separation of selection from reprogramming using lineage barcoding and paired longitudinal sampling; perturbation tests of whether lineage or niche is causally primary; operationalisation and prognostic testing of a spatial-disorder metric; subtype-annotated evaluation of KRAS-directed therapy, including exploratory lineage-shift endpoints; external validation of digital-pathology and radiomic subtype inference; and, ultimately, a prospective trial in which treatment is assigned by subtype, to convert prognostic association into predictive utility.

## 17. Conclusions

PDAC subtyping has produced one robust result—a prognostically divergent classical–basal spectrum—and one persistent limitation: the absence of validated predictive utility. Both observations are compatible with viewing subtypes as plastic cell states rather than fixed categories. This review’s contribution is not the observation of plasticity itself but its integration into a single developmental framework, linking the classical–basal spectrum to a normal-tissue reference frame, to lineage transcription factors and epigenetic remodelling, to oncogenic dosage and stromal instruction, and to treatment-induced selection or reprogramming, with spatial disorganisation proposed as a testable variable within it. The relative causal weight of these factors is unresolved, and the developmental and spatial-disorder propositions remain hypotheses requiring independent validation. If they hold, the implication extends beyond this framework: precision oncology in PDAC may depend less on assigning tumours to fixed molecular categories than on tracking how cell states move within developmental, spatial, and microenvironmental coordinates over the course of treatment—a shift from classifying tumours to charting their trajectories.

## Figures and Tables

**Table 1:** Convergence of PDAC molecular classification systems onto the classical–basal spectrum.

System (Year)	Classical-Pole Term	Basal-Pole Term	Key Contribution	Principal Limitation
Collisson [5] (2011)	Classical	Quasi-mesenchymal	First transcriptomic taxonomy	Cell-line-derived; exocrine-like class later attributed to admixture
Moffitt [6] (2015)	Classical	Basal-like	Separated tumour-intrinsic from stromal signal	Requires computational deconvolution
Bailey [7] (2016)	Progenitor	Squamous	Genomic (ICGC) validation of the two-pole axis	Immunogenic/ADEX classes largely reflect admixture
TCGA [8] (2017)	Classical	Basal-like	Demonstrated that purity drives spurious classes	Small, purity-selected cohort
Puleo [9] (2018)	Classical	Basal-like	Integrated a separable stromal axis	Tumour and stromal signal not fully disentangled
Chan-Seng-Yue [10] (2020)	Classical	Basal-like	First single-nucleus resolution; hybrid tumours	Finer sub-splits poorly reproducible
PurIST [11] (2020)	Classical	Basal-like	Purity-independent single-sample classifier	Binary output omits intermediate biology

**Table 2:** Classical versus basal-like states: a concise comparison.

Feature	Classical	Basal-Like
Defining regulators	GATA6, HNF1A, HNF4A, PDX1	ΔNp63 (TP63)
Representative markers	TFF1–3, REG4	KRT5/6/17, S100A2
KRAS dosage	Lower	Higher (interpretation contested)
Prognosis	Better	Worse (stage-confounded)
Chemotherapy association	Better observed outcomes	Poorer observed outcomes

**Table 3:** Translational status of the classical–basal framework, by clinical domain. Established findings, current limitations and proposed future research are presented in separate columns and do not represent equivalent levels of evidence; entries in the final column are research proposals requiring validation, not current practice.

Domain	What Is Established	Current Evidence	Current Limitation	Future Direction (Proposed Research, Not Current Practice)
Prognosis	Basal-like state and low GATA6 associate with worse outcome	COMPASS [21]; GATA6 IHC cohorts [35]; real-world subtyping [39]	Confounded by stage and resectability	Stage-adjusted models; spatial-disorder metric as adjunct
Prediction	No validated predictive biomarker	GemPred (retrospective) [40]; PASS-01 (randomised; treatment not assigned by subtype) [41]	No completed trial has assigned treatment by subtype	Prospective subtype-stratified/adaptive trials
Assays	Bulk classifiers are purity-confounded	PurIST [11]; PAMG [42]; GATA6 immunohistochemistry [43]	No assay has demonstrated clinical utility	Purity-independent, longitudinally repeated assessment
Surgery	Subtype should not alter surgical selection outside a trial	Neoadjuvant pathways enable paired, multi-region sampling [14,39]	Diagnostic material increasingly small and stroma-rich	Embed subtyping into surgical/neoadjuvant trials with tissue banking
KRAS-directed therapy	KRAS is now pharmacologically targetable	Preclinical G12D-selective inhibitors active in PDAC models [44,45]	Whether subtype predicts response is unresolved	Subtype-annotated correlatives in RAS-inhibitor trials
Spatial biology	Classical and basal programmes are spatially intermixed, not segregated	Intra-ductal heterogeneity [23]; ordered domains in adenosquamous carcinoma [27]	No validated spatial-disorder metric	Operationalise and prognostically test a spatial-disorder metric
Digital pathology/AI	Manual IHC scoring has limited reproducibility	Computer-assisted GATA6 scoring [43]; combination IHC panels [46]	Radiomic/pathomic inference remains early-stage	External validation of digital-pathology classifiers

## Data Availability

No new data were created or analyzed in this study. Data sharing is not applicable to this article.

## Abbreviations

The following abbreviations are used in this manuscript:

ADEX	Aberrantly differentiated endocrine–exocrine
CAF	Cancer-associated fibroblast
CRT	Chemoradiotherapy
ctDNA	Circulating tumour DNA
DFS	Disease-free survival
IHC	Immunohistochemistry
KRAS	Kirsten Rat Sarcoma Viral Oncogene Homologue
NAPOLI-3	NAnoParticle albumin-bound paclitaxel plus Oxaliplatin, Leucovorin and Irinotecan trial
OS	Overall survival
PAMG	Pancreatic adenocarcinoma molecular gradient
PASS-01	Pancreatic Adenocarcinoma Signature Stratification for Treatment-01
PDAC	Pancreatic ductal adenocarcinoma
PurIST	Purity-independent subtyping of tumours
RCT	Randomised controlled trial
TCGA	The Cancer Genome Atlas
WGS	Whole-genome sequencing
